# Lunatic Asylums

**Published:** 1846-06

**Authors:** 


### ARTICLE XI.

#### LUNATIC ASYLUMS.

We have received the twenty-fifth annual report of the Bloomingdale Asylum for the Insane, for the year 1845. By Pliny Earle, M. D., Physician to the Asylum. Also the third annual report of the Managers of the New York State Lunatic Asylum, made to the Legislature, Jan. 23, 1846; comprising also the report of the Superintendant of the Institution, Amariah Brigham, M. D.

Both of these interesting papers contain much to cheer us with the hope that the praiseworthy efforts of those who are devoting their talents and lives to the alleviation and cure of Insanity, are fast acquiring the means of controlling this once most formidable disease, to an extent beyond the expectations of the most enthusiastic advocates of modern treatment.

As appears from the report of the Bloomingdale Asylum, the whole number in that Institution during the year 1845, was 242. Of this number, 125 were discharged during the time, 61 cured, 12 much improved, 20 improved, 20 unimproved, 12 died. In the New York State Lunatic Asylum the total number in the course of the same year, was 553. Discharged recovered 135, improved 78, unimproved 34, died 21. Total number discharged during the year 268.

In the report of Dr. Earle, we find the following important facts and conclusions:

"First. *As a general rule, the first measure in the curative treatment of Insanity, is to remove the patient from home, from acquaintances, and from all familiar scenes and associations.*

"Second. *When the insane are placed under the proper curative treatment in the early stages of the disease, from* 75 *to* 90 *per cent recover.*

"Third. *On the contrary, if they be not put under treatment before the disease has continued a year or more, from* 15 *to* 20 *per cent only, are cured.*"

In the report of Dr. Brigham of the New York State Lunatic Asylum, are some very judicious remarks upon the "neglect of the study of insanity, by physicians," which we place among our selections, with the view of directing the attention of our readers to this important subject. W. B. H.

